# Editorial: A holistic approach to adolescent oral, mental, and sexual wellness

**DOI:** 10.3389/froh.2025.1727730

**Published:** 2025-11-25

**Authors:** Moréniké Oluwátóyìn Foláyan, Bibilola Damilola Oladeji

**Affiliations:** 1 Department of Child Dental Health, Obafemi Awolowo University, Ile-Ife, Nigeria; 2 Department of Psychiatry, University of Ibadan, Ibadan, Nigeria

**Keywords:** adolescent health, integrated health services, co-creation, health equity, common risk factor approach, oral health, mental health, sexual and reproductive health

Adolescence is a pivotal, turbulent, and opportunity-rich period of human development that deserves respect and understanding (1). It is a time when the trajectories of lifelong oral, mental, sexual, and reproductive health are set, for better or worse (2–4). Characterized by profound physical, psychological, and social changes, this phase sees young people navigating a complex web of influences, from the push for peer acceptance to the formation of personal identity (5), all of which shape health behaviours. Currently, 10%–75% adolescents have oral health problems (6), one in seven experiences a mental disorder (7), and 50% of the 21 million adolescent pregnancies each year are unwanted, with 55% of these in low- and middle-income countries end in abortion, many of which are unsafe (8). This Research Topic was founded on the conviction that these health domains are not siloed but are deeply intertwined (9). In an era defined by digital connectivity and global challenges, understanding these interconnections requires us to also consider the new online and offline landscapes that adolescents navigate. The collection of 11 manuscripts published within this topic provides a critical evidence base for this holistic view.

The publications provide evidence that health challenges in one area often cascade into others. The link between mental and oral health is clearly established: Geraets and Heinz demonstrate that adolescents with higher stress and lower life satisfaction are less likely to engage in regular toothbrushing, while Lawal et al.'s qualitative work reveals that adolescents themselves view good oral health as crucial for confidence and social interaction. This mind-body connection extends to reproductive health; Liu et al. show that academic stress is a significant factor in dysmenorrhea and subsequent school absenteeism.

Furthermore, reproductive health issues are never merely physical. Studies from Kenya (Mason et al.) and China (Liu et al.) highlight their profound psychosocial impact. The “fear, responsibility, and blame” associated with unintended pregnancy and the desperate lengths taken to secure unsafe abortions create significant mental distress, disrupting education and social life. Although not discussed by the collection of manuscripts, we must recognize the digital environment as a primary determinant of adolescent wellness. Social media can exacerbate body image issues and cyberbullying, directly impacting mental health. Simultaneously, online platforms provide both dangerous misinformation on topics like contraception and vital peer support. Digital literacy and the ethical deployment of digital health tools must become integral to modern adolescent health strategies.

More importantly, the manuscripts move beyond diagnosis to outline an integrated approach, pairing key challenges with evidence-based solutions. Kyei-Baafour et al. and Hlaing et al. demonstrate that factors like parental education, wealth, and residential location shape everything from hormone levels to sexual behaviors. Thus, foundational policies aimed at poverty alleviation and educational empowerment are non-negotiable for any integrated health strategy. This is more so that parents, like those who live in Canada (Punjani et al.), feel ill-equipped to provide sexuality education, while adolescents in Kenya (Mason et al.) hold dangerous misconceptions. Wang et al. showed that “good” school-based sex education improves knowledge and safer behaviors. This must be complemented by co-designed, culturally attuned resources for parents, as Punjani et al. recommend.

In addition, authors identified that stress from academics (Liu et al.) or general life (Geraets and Heinz) impairs self-care and exacerbates conditions like dysmenorrhea. Also, gendered power dynamics and stigma, as seen in Kenya (Mason et al.), increase vulnerability for girls. Song et al.'s meta-analysis, however, prescribes aerobic exercise as a non-stigmatizing intervention for anxiety and depression. Community-level work to address harmful norms is equally essential. Although none of the manuscripts addressed oral, mental sexual and reproductive health issues among vulnerable populations, it is important to recognise that LGBTQ+ adolescents and other marginalized groups face heightened health disparities due to stigma, discrimination, and a lack of inclusive services (10). This underscores the necessity of meaningful co-creation, ensuring interventions are designed with and for diverse youth, to ensure that services developed are truly accessible and acceptable to all.

Importantly, the collection offers concrete tools for this integration. Afolabi et al. developed a holistic assessment tool to identify co-morbidities, while the Teen Research Advisors (Tumberger et al.) program provides a blueprint for youth engagement. At the systems level, change is paramount. Lawal et al. advocate for health promotion in schools, ideal for integrating messages on mental, oral, and reproductive health. Findings from Kenya (Mason et al.) and Myanmar (Hlaing et al.) underscore the need for policy interventions to ensure access to youth-friendly services.

The next critical step is to address scalability and sustainability by moving from successful pilot studies to the integration of these approaches into under-resourced public health and education systems. This can happen through the meaningful co-creation of programs, by sharing power and decision-making with adolescents to avoid tokenism and ensure relevance.

In conclusion, this research topic provides a robust platform, demonstrating that effective adolescent health strategies must be holistic, rooted in a common risk factor approach, co-created with youth, and multi-sectoral. Addressing the complex needs of adolescents today demands strategies that are holistic and agile enough to respond to a rapidly changing world. This means acknowledging the digital determinants of health, prioritizing the global adolescent oral, mental, sexual, and reproductive health crisis, and ensuring solutions are equitable and inclusive for all young people, regardless of their background or identity. The evidence is here; the imperative now is to act with collaboration, urgency, and a commitment to building a future where every adolescent can thrive.
